# Micro-inflammation related gene signatures are associated with clinical features and immune status of fibromyalgia

**DOI:** 10.1186/s12967-023-04477-w

**Published:** 2023-09-05

**Authors:** Menghui Yao, Shuolin Wang, Yingdong Han, He Zhao, Yue Yin, Yun Zhang, Xuejun Zeng

**Affiliations:** Division of General Internal Medicine, Department of Family Medicine, Peking Union Medical College Hospital, Chinese Academy of Medical Sciences, State Key Laboratory of Complex Severe and Rare Diseases (Peking Union Medical College Hospital), Beijing, 100730 China

**Keywords:** Fibromyalgia, Micro-inflammation, Depression, Dendritic cells

## Abstract

**Background:**

Fibromyalgia (FM) is a multifaceted disease. Along with the genetic, environmental and neuro-hormonal factors, inflammation has been assumed to have role in the pathogenesis of FM. The aim of the present study was to explore the differences in clinical features and pathophysiology of FM patients under different inflammatory status.

**Methods:**

The peripheral blood gene expression profile of FM patients in the Gene Expression Omnibus database was downloaded. Differentially expressed inflammatory genes were identified, and two molecular subtypes were constructed according to these genes used unsupervised clustering analysis. The clinical characteristics, immune features and pathways activities were compared further between the two subtypes. Then machine learning was used to perform the feature selection and construct a classification model.

**Results:**

The patients with FM were divided into micro-inflammation and non-inflammation subtypes according to 54 differentially expressed inflammatory genes. The micro-inflammation group was characterized by more major depression (*p* = 0.049), higher BMI (*p* = 0.021), more active dendritic cells (*p* = 0.010) and neutrophils. Functional enrichment analysis showed that innate immune response and antibacterial response were significantly enriched in micro-inflammation subtype (*p* < 0.050). Then 5 hub genes (*MMP8*, *ENPP3*, *MAP2K3*, *HGF*, *YES1*) were screened thought three feature selection algorithms, an accurate classifier based on the 5 hub DEIGs and 2 clinical parameters were constructed using support vector machine model. Model scoring indicators such as AUC (0.945), accuracy (0.936), F1 score (0.941), Brier score (0.079) and Hosmer–Lemeshow goodness-of-fit test (χ^2^ = 4.274, *p* = 0.832) proved that this SVM-based classifier was highly reliable.

**Conclusion:**

Micro-inflammation status in FM was significantly associated with the occurrence of depression and activated innate immune response. Our study calls attention to the pathogenesis of different subtypes of FM.

**Supplementary Information:**

The online version contains supplementary material available at 10.1186/s12967-023-04477-w.

## Introduction

Fibromyalgia (FM) is a disease characterized by chronic widespread muscle and skeletal pain, often accompanied by fatigue, non-restorative sleep, cognitive impairment, depression and anxiety [1]. In terms of prevalence, FM has been the third most common musculoskeletal disease, after lumbar pain and osteoarthritis. Epidemiological surveys showed that the prevalence of FM in the global population can be as high as 2.7%. and female patients are far more than males [2, 3]. Not all clinicians are familiar with the clinical characteristics of FM, leading to its underdiagnosis. Central sensitisation was thought to be the key driver of FM, and genetic, environmental, and hormonal factors are also involved [4]. However, these theories were still insufficient to explain the pathogenesis of FM.

In recent years, some researchers identified the existence of a previously underappreciated subtype of FM, called inflammatory FM [5]. It has higher levels of some inflammatory markers/mediators, cytokines, and glycoproteins compared to control group, suggesting that these elements could be important factors that may contribute to the onset of inflammatory FM [6]. Therefore, more and more researches focused on the immunological alterations and inflammation background of FM. Kadetoff et al. found the concentration of IL-8 were increased in the cerebrospinal fluid of FM patients, which could be due to the activation of glial cells [7]. In addition to this, some studies have shown an increase in serum concentrations of IL-6, IL-8, IL-1β and TNF-α in patients with FM [8–11]. These findings support the hypothesis that there is a low-grade systemic activation of immune-inflammatory pathways in FM, however, no clear correlation in these studies has been identified between symptom severity and these cytokines. Some immune cells such as mast cells, monocytes and neutrophils were speculated that they might play a role in the formation of the inflammatory matrix of fibromyalgia, but the specific mechanism was still unclear [12]. Animal experiments found resident macrophages located in the muscle and pro-inflammatory cytokines, such as interleukins (IL-1β, IL-6) might contribute to the development of chronic widespread muscle pain [13]. Most of these researches have remained at the serological levels, we still know very little about the role of inflammation in FM pathogenesis. Where does the inflammation come from? Does it relate to comorbid conditions? How do we distinguish inflammatory FM?

The combination of information technology and molecular biology has led to the emergence of bioinformatics, which has been used to reinterpret disease at the gene levels. However, there are few studies on bioinformatics in FM. Lukkahatai et al. performed peripheral blood RNA sequencing from 9 women with FM. Differentially expressed genes (DEGs) between high and low Pain Catastrophising Scale scores revealed functional pathways associated with interferon signaling, interferon regulatory activation and dendritic cell maturation were significantly dysregulated [14]. Jones et al. analyzed RNA expression in FM patients and healthy controls, and they found DEGs were associated with the pathways for pain processing, such as glutamine/glutamate signaling and axonal development. They also found several inflammatory pathways were up-regulated, but further research was not conducted [15].

Here, we obtained the peripheral blood gene expression profiles of FM patients from the GSE67311 dataset. Differentially expressed inflammatory genes (DEIGs) were further identified, and two molecular subtypes were constructed according to these genes used unsupervised clustering analysis. The clinical characteristics, immune features and pathways activities were compared further between the two subtypes. We used rigorous feature selection strategies—five hub genes were “locked” and constructed a classification model by Support Vector Machine (SVM) combined 2 clinical parameters. Our study is the first to identify the different inflammatory subtypes and may provide new insights into the pathogenesis of inflammatory FM.

## Methods

### Patients and samples

The peripheral blood gene expression profiles of GSE67311 from FM patients and healthy controls were downloaded from the Gene Expression Omnibus (GEO). GSE67311 is a microarray dataset generated by the Affymetrix GeneChip Human Genome HG-U133A Custom CDF. It included 142 samples from 61 FM patients and 68 normal controls. Then, the annotation document of corresponding platforms was used to annotate the gene expression profiling in each dataset. Finally, the matrix with row names as sample names and column names as gene symbols was obtained for subsequent analysis. In addition, the clinical characteristics of FM patients were also extracted at the same time.

### Identify the differentially expressed inflammatory genes

We used the packages “limma” in R software 4.0.0.0 to performed DEGs analysis between the case and normal group on GSE67311. The *p*-value < 0.05 was considered as DEGs. Volcano plot was used to reveal the expression patterns of these DEGs. Then, the inflammatory genes in the Gene Ontology Term (GO0006954: inflammatory response) were obtained. The overlapped genes in DEGs and inflammatory genes were obtained using Jvenn, and these genes were defined as DEIGs.

### Consensus clustering analysis based on differentially expressed inflammatory genes

To explore the influence of the inflammatory genes on the FM, cluster analysis was performed based on the mRNA expression profiles of DEIGs using the kmdist clustering in the R packages “ConsensusClusterPlus”. The optimal number of clusters was determined according to intragroup consistency. The clinical features of subtypes were then compared to explore the differences between two subtypes.

### Single-sample gene-set enrichment analysis

Single-sample gene-set enrichment analysis (ssGSEA) is an algorithm used to calculate the relative activity of each pathway or immune cells based on the gene expression levels of a single sample, and the calculation was completed using the R packages “GSVA” and “GSEABase” [16]. In the study, we used the ssGSEA to quantify the inflammatory response scores of each FM patients and the infiltration scores of 28 immune cells. For the inflammatory response, the gene signature was obtained from the Molecular Signatures Database (C5: GO gene sets). For the immune infiltration, the gene set for marking 28 immune cell types was enrolled from a previously published article (see Additional file 1: Table S1) [17].

### Functional enrichment analysis

Gene Ontology (GO) analysis was used to describe the attributes of genes and gene products, including molecular function (MF), biological process (BP) and cellular component (CC). The Kyoto Encyclopedia of Genes and Genomes (KEGG) and Reactome pathways enrichment analysis was used to obtain pathways at the gene level [18].

Similarly, we used the “limma” packages to identify the DEGs between the two subtypes of FM and set the appropriate thresholds (log_2_foldchange > 0.4 or < -0.4, *p*-value < 0.05) to screen for the suitable number of DEGs, we then performed the GO BP, KEGG and Reactome pathways analyses via DAVID website.

### Feature selection and construction of classification model

Least Absolute Shrinkage and Selection Operator (LASSO) is a linear regression method that uses L1 regularization to shrink some unimportant coefficients to zero [19]. Random Forest Recursive Feature Elimination (RF-RFE) is a feature selection method that uses Random Forests to evaluate the importance of each feature and recursively removes the least important features until a desired number of features are left [20]. eXtreme Gradient Boosting (XGBoost) is a gradient boosting algorithm that uses decision trees as base learners. It is widely used in data mining, feature selection, and other fields [21]. Therefore, we performed the LASSO, RF-RFE, and XGBoost algorithm using “glmnet”, “randomForest”, and “xgboost” packages in R software separately to reduce the dimensions of the gene expression matrix.

SVM is a powerful supervised learning algorithm used for classification or for regression developed by Cortes and Vapnik in 1995 [22]. The key to algorithms is to find a hyperplane that best divides a dataset into different classes. To achieve linear separability of the samples, SVM constructed a mapping function that maps nonlinear features to a new high-dimensional space. The kernel function is used to simplify the inner product operation in the mapping space and ensure that the results are equivalent [23]. In this study, the SVM algorithm is performed using “e1071” package and the kernel function using the Radial Basis Function, other parameters are default.

### Model test and evaluation

The whole data was randomly (random seed:100) divided into two equal parts using the “e1071” package: one was used to train the SVM model, and the other was used for internal validation. The indicators, comprising area under the receiver operating characteristic (ROC) curve (AUC) value, accuracy, F1_score, and Brier score, were used to evaluate the SVM model. The Brier score is a proper score function that quantifies the accuracy of probabilistic predictions. It is applied to tasks in which predictions must assign probabilities to a set of mutually exclusive discrete outcomes. The closer the score is to zero, the more accurate the model [24]. At the same time, Hosmer–Lemeshow goodness-of-fit test was used to evaluate the calibration performance of the model, *p-*value > 0.05 indicated a great calibration performance [25].

### Statistical analysis

Statistical software SPSS 25.0 (IBM Corporation, USA) was used for statistical analysis. Chi-square test was used for qualitative data, students’t-test was used for quantitative data between two groups. One-way ANOVA was used for three groups, who’s the *p*-value was adjusted by BH method. Correlation analysis was performed by Pearson’s linear correlation. The *p*-value < 0.05 was considered as statistically significant.

## Results

### Inflammation-related DEGs were identified in the GEO cohort

**Fig. 1:**
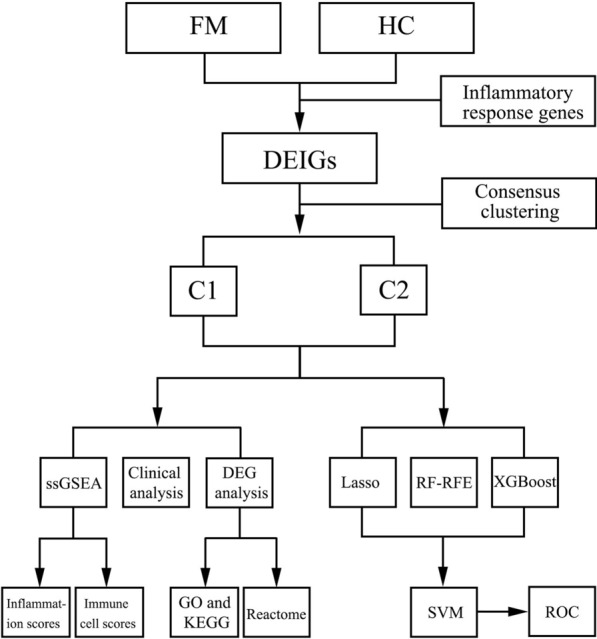
Work flow of this study

**Fig. 2:**
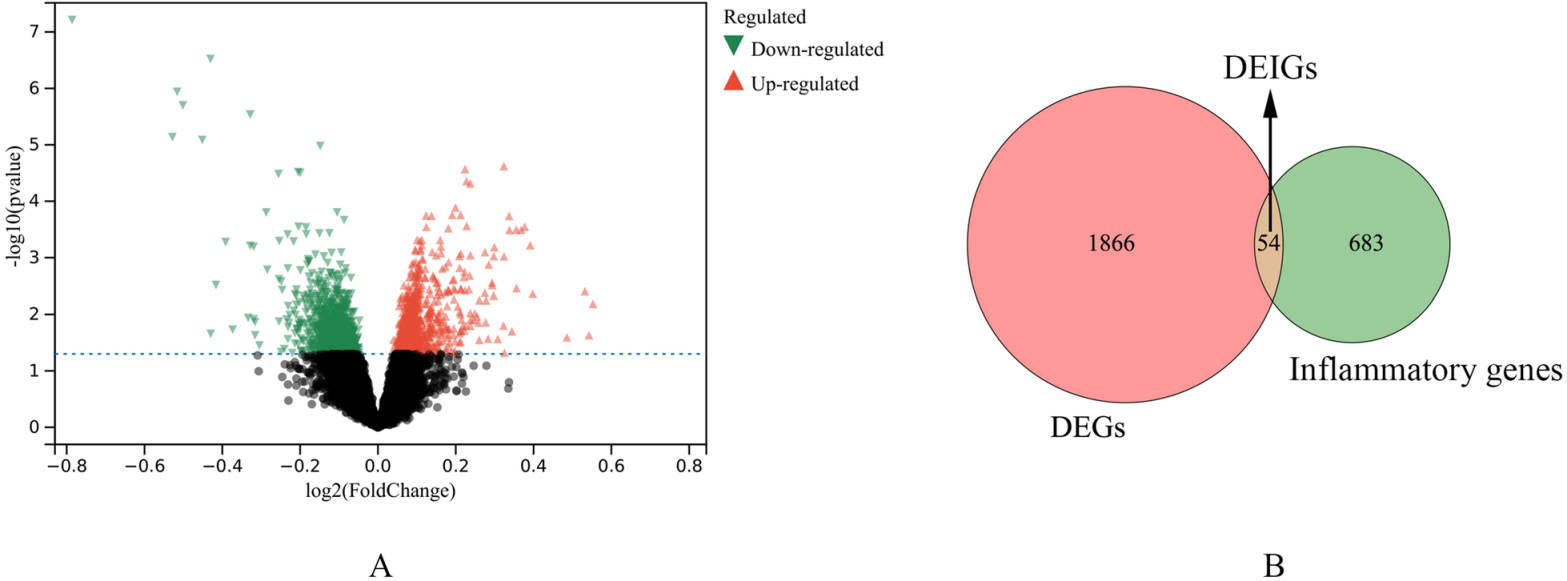
Identification of the DEIGs. **A** The volcano plot of DEGs between FM patients and control group. **B** The Venn diagram of the DEIGs

The flow chart of the whole analysis is shown in Fig. 1. A total of 1920 DEGs were identified between the FM patients and the control group, including 960 up-regulated DEGs and 958 down-regulated DEGs, and they were shown as volcano plot (Fig. 2A) (Additional file 1: Table S2). 774 inflammatory genes were obtained from GO 0006954 term: inflammatory response, 54 overlapped genes were obtained (Fig. 2B) (Additional file 1: Table S3).

### Patients were stratified into different inflammation status though consensus clustering

**Fig. 3:**
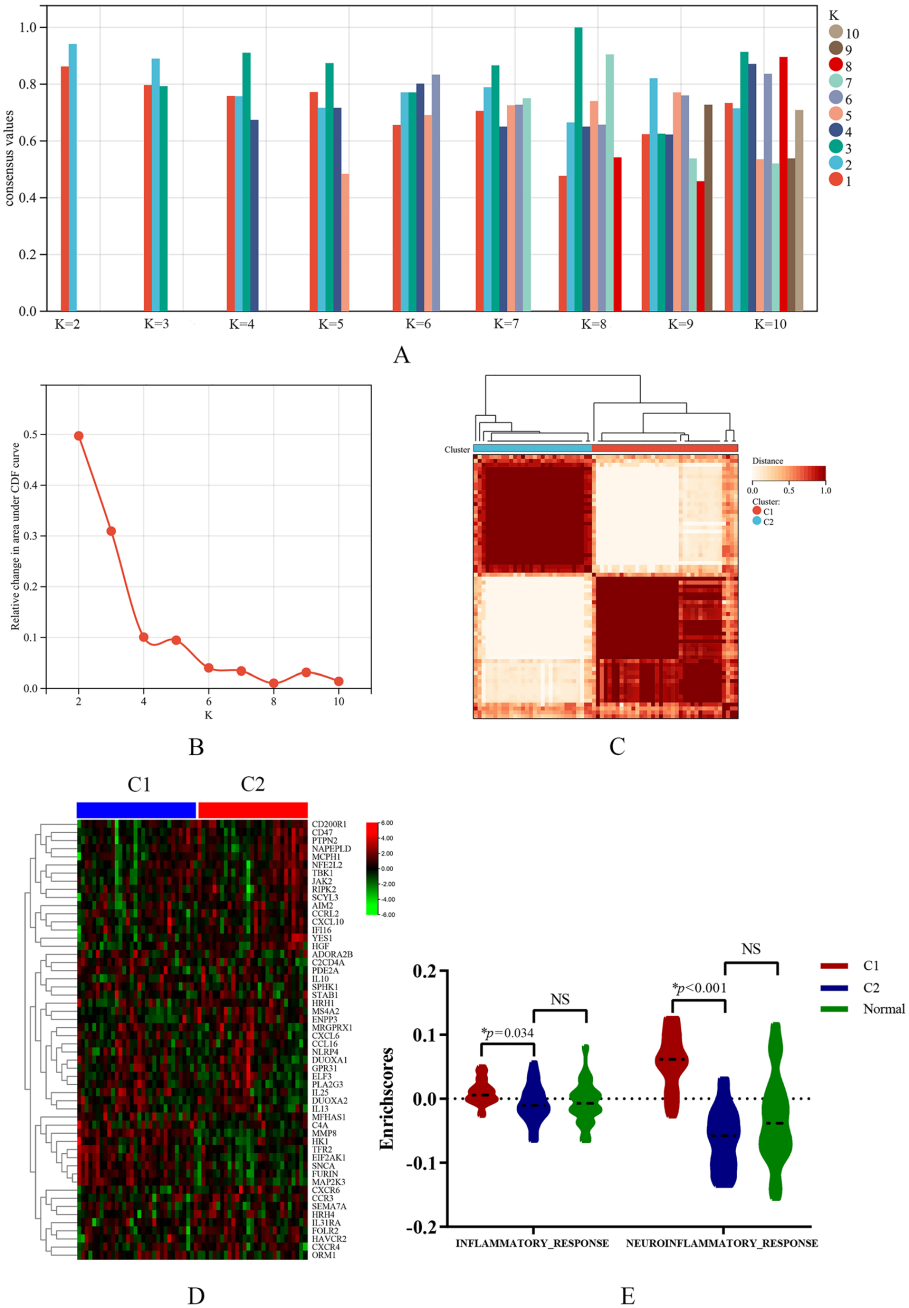
Consensus clustering analysis. **A** The bar plot representing the cluster consensus scores for different numbers of clusters (K ranges from 2 to 10) for patients with FM. When K = 2, both the cluster consensus scores were greater than 0.8, representing the higher stable. **B** Delta area plot reflecting the relative changes in the area under the CDF curve from k to k-1. **C** Consensus matrix heatmap of FM microarray samples at k = 2, the samples are clearly clustered into two clusters. Therefore K = 2 was identified the optimal value for consensus clustering. **D** Heatmap visualizing the expression of DEIGs in the two subtypes in FM patients. **E** Violin plots indicating the differences of inflammatory response scores among the C1, C2 and control group. * *p*-value was adjusted by Bonferroni method. *CDF* cumulative distribution function

Based on the mRNA expression profiles of 54 DEIGs, 61 FM patients were classified into the two molecular subtypes (C1: n = 31; C2: n = 30) by unsupervised clustering analysis after deleting duplicate samples (Fig. 3A–C). The whole expression profiles of the DEIGs were different between two subtypes (Fig. 3D). We further assessed the inflammatory response enrichment scores of each sample using ssGSEA algorithm. The results showed that the inflammatory response in C1 group were higher than that in C2 and normal group, especially in their child term, neuroinflammatory response (Fig. 3E). Due to the small Fold-changes of the DEIGs, the C1 group were defined as the micro-inflammation subtype, and the C2 group were defined as the non-inflammation subtype.

### Different inflammation status presented variant clinical characteristics, immune features and pathways activities in FM

The clinical characteristics of the two subtypes were further compared in Table 1. The proportion of patients with major depression were significantly increased in the micro-inflammation group, indicating that the micro-inflammation may be related to the occurrence of depression The BMI were also increased in micro-inflammation group. However, the Fibromyalgia Impact Questionnaire Revised (FIQR) scores did not have statistically significant (Table 1).

**Table 1:** The clinical characteristics of two subtypes in FM

	C1	C2	χ^2^/t	*p-*value
Total patients	31	30		
Irritable bowel syndrome, n (%)	14(45.1%)	16(53.3%)	0.407	0.523
Major depression, n (%)	17(54.8%)	9(30.0%)	3.846	**0.049**
Bipolar disorder, n (%)	1(3.1%)	2(6.6%)	0.001	0.997
Chronic fatigue syndrome, n (%)	4(12.9%)	7(23.3%)	1.122	0.289
Migraine, n (%)	17(54.8%)	13(43.3%)	0.807	0.369
BMI, mean (SD)	34.71(6.74)	30.70(6.58)	2.377	**0.021**
FIQR, mean (SD)	50.05(16.91)	58.37(17.14)	− 1.941	0.057

*BMI* body mass index, *FIQR* The Revised Fibromyalgia Impact Questionnaire

**Fig. 4:**
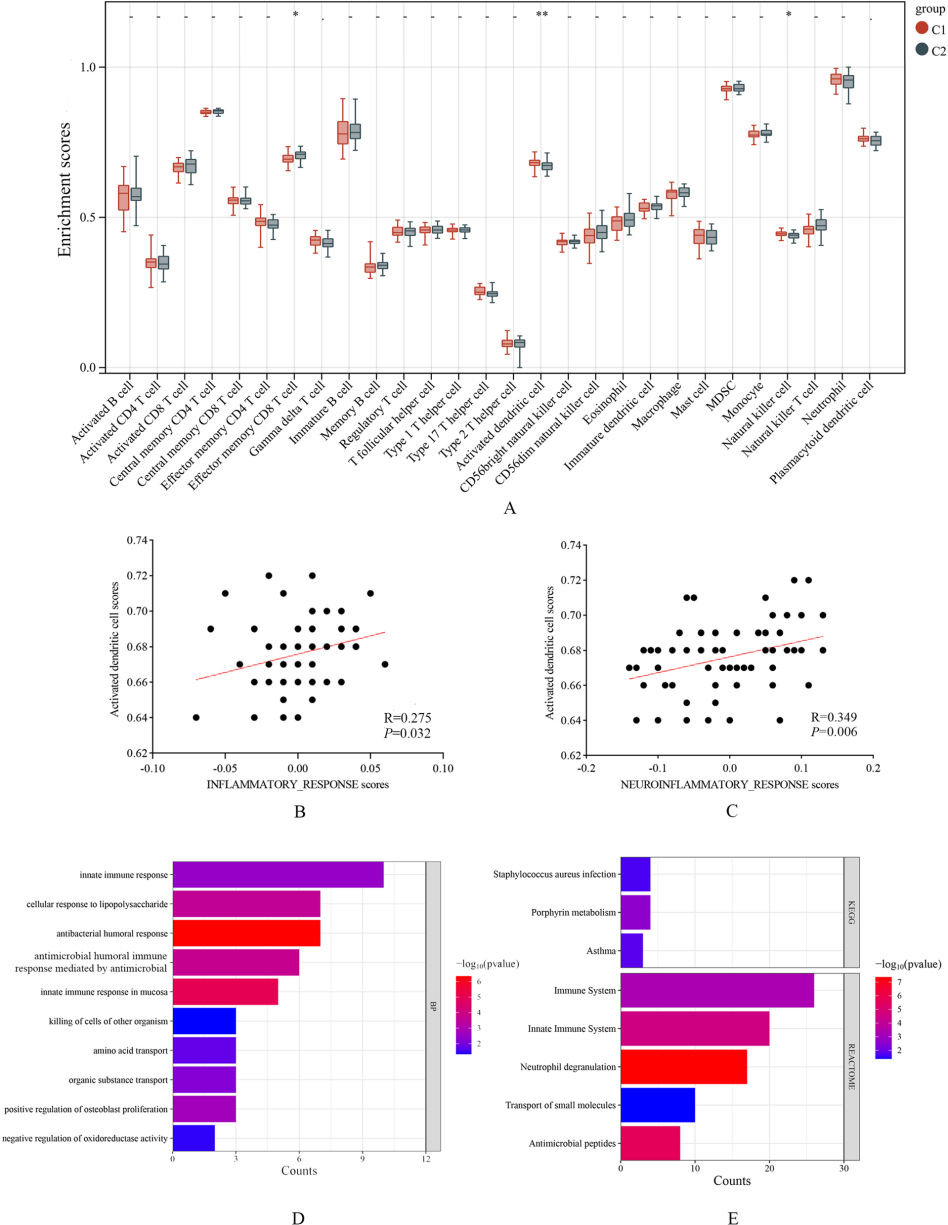
Immune infiltration and functional enrichment analyses. **A** The scores of 28 immune cell types between the two subtypes in FM patients. **B** The relationship between activated dendritic cells scores and inflammatory response. **C** The relationship between activated dendritic cells scores and neuroinflammatory response scores. **D** The partial terms of GO BP. **E** The partial terms of the KEGG and REACTOM pathways

Furthermore, we quantified the enrichment scores of immune cells in the blood of two subtypes used ssGSEA algorithm. Compared to the non-inflammation subtype, the fraction of activated dendritic cells and natural killer cells were higher in micro-inflammation subtype, but the effector memory CD8 T cell was higher in the non-inflammation subtype (Fig. 4A). We further assessed the correlation between dysregulated immune cells scores and inflammation response scores, and we found activated dendritic cells were positively activated dendritic cells were positively correlated with inflammatory response and neuroinflammatory response (Fig. 4B, C), indicating that the micro-inflammation in FM may be related activated dendritic cells.

We further performed the DEG analysis between the micro-inflammation and non-inflammation subtypes, and a total of 106 DEGs were identified (Additional file 1: Table S4). GO BP analysis showed some immune responses were enriched, including the innate immune response, antimicrobial immune response, and cellular response to lipopolysaccharide. Besides, some response relating to cell killing and biological regulation were also enriched (Fig. 4D). KEGG analysis showed some disease-related signaling pathways were significantly enriched, including transcriptional misregulation in cancer, *Staphylococcus aureus* infection, asthma. Based on the similarity of their gene disorders, therefore, the pathological mechanisms of the micro-inflammation subtype in FM may overlap with that in some immune and infectious diseases. Enriched Reactome pathways were similar with GO BP terms, including the immune response process, neutrophil dysregulation and antimicrobial peptides (Fig. 4E).

### Multiple machine learning algorithms were used to develop the molecular subtype classifier

**Fig. 5:**
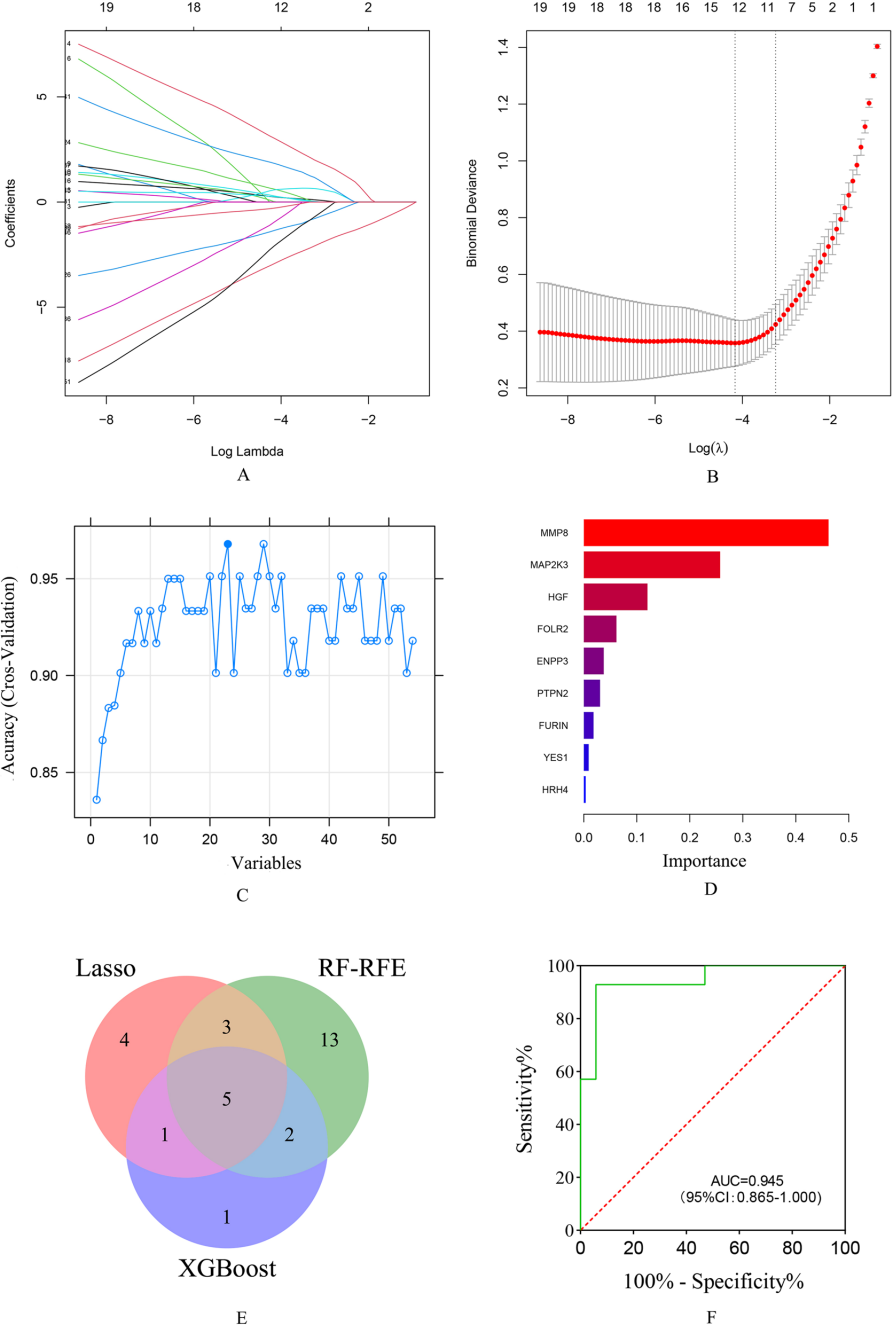
Feature selection and SVM model. **A** LASSO coefficient profiles of candidate DEIGs. The LASSO was used for regression of high dimensional predictors. The method uses an L1 penalty to shrink some unimportant regression coefficients to exactly zero. **B** Cross-validation to select the optimal tuning parameter log (Lambda) in LASSO regression analysis. The left dotted line represents the minimum error of the model, while the right dotted line represents the minimum number of features of the model within the range of error tolerance. In our study, we chose the model with the minimum error. **C** Performance measure of the cross-validated RF-RFE technique. When there are 23 features(variables), the model has the highest accuracy. **D** Features’ importance ranking in XGBoost analysis, a longer bar represented the variables had more influence on the outcome variables. **E** Venn diagram showing the hub genes shared by LASSO, RF-RFE, and XGBoost algorithm. **F** The ROC curve of the internal validation using SVM model

We aimed to find core inflammatory subtype relevant features based on 54 DEIGs to establish a classifier that could specifically predict the different subtype of FM patients. 3 machine learning algorithms were used to perform feature selection, a total of 13(*HGF*, *YES1*, *MS4A2*, *HRH4*, *MMP8*, *HRH1*, *MAP2K3*, *ENPP3*, *CCR3*, *IL13*,*ORM1*, *IL10*, *SNCA*), 23(*MMP8*, *MAP2K3*, *FURIN*, *EIF2AK1*, *ENPP3*, *HRH1*, *HG*F, *MS4A2*, *MCPH1*, *TFR2*, *SNCA*, *YES1*, *HK1*, *CD200R1*, *C2CD4A*, *ORM1*, *C4A*, *PLA2G3*, *IL10*, *STAB1*, *FOLR2*, *NAPEPLD*, *CD47*), 9(*MMP8*, *ENPP3*, *MAP2K3*, *FURIN*, *HGF*, *HRH4*, *PTPN2*, *FOLR2*, *YES1*) DEIGs were selected using Lasso, RF-RFE, and XGBoost algorithms, separately(Fig. 5A–D). 5 hub DEIGs were obtained after the intersection, including *MMP8*, *ENPP3*, *MAP2K3*, *HGF*, *YES1* (Fig. 5E). In addition, clinical features such as depression and BMI were significantly different between the two subtypes. We theorize that these features may also have pertinent classification values in FM. Therefore, we applied SVM model to construct a classifier for different inflammation sub*t*ype, based on the 5 hub DEIGs and 2 clinical parameters, depression and BMI. The optimal threshold for classification was 0.421, which meant that if a patient with a probability > 42.1%, he or she would be classified into micro-inflammation subtype, else he or she would be classified into non-inflammation subtype.

We performed ROC curve analysis on the internal testing set to evaluate the model and found that the area under curve (AUC) reached 0.945 with a sensitivity of 0.941 and a specificity of 0.929. Besides, the accuracy in the model was 0.936, F1 score was 0.941, brier score was 0.079, indicating that this SVM-based classifier was highly reliable. While the Hosmer–Lemeshow goodness-of-fit test showed that our predicted and observed values were close (χ^2^ = 4.274, *p* = 0.832), suggesting a great calibration performance.

## Discussion

FM is a chronic disease with generalized diffuse pain as the main clinical feature. Its pain is diverse in nature and difficult to relieve after rest. Symptoms include depression, anxiety, fatigue, decreased sleep quality and cognitive dysfunction, which seriously affects the quality of life and social function of patients [1]. FM was originally classified as a non-inflammatory disease, but, over the past years, extensive researches has been implicit that the inflammation might be associated with FM closely. The up-regulation of the inflammatory cytokines such as IL-1, IL-8 and TNFα were also found to be associated with the disease-related comorbidities [26]. Andrade et al. reported there was a low-grade chronic inflammation state in FM [27]. The small fold-change of genes in our study, which cannot be used to identify DEGs between two subtypes, also confirmed the above findings. Although the expression levels of DEIGs were very similar between the non-inflammation and micro-inflammation subtypes, statistical significance remained evident, and ssGSEA analysis showed the inflammation level in C1 subtype was higher. Small differences in gene expression levels may cause dramatic differences in clinical phenotypes. Based on our results, we try to answer following questions.

### Does inflammation relate to comorbid conditions?

FM is also comorbid with a variety of psychiatric disorders, including depression, anxiety disorders, and so on. Some studies have shown that 25 to 30% of FM patients were diagnosed with severe depression [28], while the lifetime prevalence of depression in FM is 74% [29]. Interestingly, we found that compared to the non-inflammation subtype, the micro-inflammation subtype had more depression, and the FIQR didn’t have significant difference. The mechanisms of depression in FM are still unclear. Some studies reported that the largely overlapping pathophysiological processes processed mood and pain in the brain might result in the high rates of FM and mood disorder comorbidity [2, 30]. Our analysis showed that elevated inflammation level may be related to the occurrence of depression in FM. In fact, it has been observed that chronic inflammation increased incidence of “comorbid” depressive symptoms [31, 32]. Elevated levels of peripheral inflammation were reported to cause depression. The underlying mechanism may be involved in the changes of micro-structural and functional connectivity in the default mode network of the brain [33]. Studies using positron emission computed tomography imaging with the 18 kDa translocator protein as a biomarker of microglia also have demonstrated that neuroinflammation exists in multiple brain regions in patients with depression [34]. Therefore, the persistent state of micro-inflammation in FM may be one of the main causes of depression. For patients with FM and depression, combined anti-inflammatory treatment with antidepressant treatment may be a new therapeutic strategy.

Another clinical feature in our study was that most of the FM patients have a high-level BMI, and the micro-inflammation subtype was significant higher. Many studies have reported that FM patients tend to be overweight when compared with general population, and they have lower quality of life and higher pain sensitivity [35, 36]. Recent evidences suggested that overweight and obesity may be characterized by a low-grade chronic inflammatory state, as reflected by elevated levels in inflammatory mediator such as high sensitivity C-reactive protein (CRP) [37]. Our results also supported the notion that obesity in FM, as measured by BMI, may be a clinical marker for increased levels of inflammation.

### Where does inflammation come from?

Immune analysis showed the proportions of activated dendritic cells and nature killer cells (NK cells) were significantly increased in micro-inflammation subtype, but the effector memory CD8 + T cells were decreased. We further assessed the correlation among the immune cells’ infiltration scores and inflammatory response scores, and we found that the activated dendritic cells were positively correlated with inflammatory response and neuroinflammatory response. Dendritic cells (DCs) are a specialized subset of antigen presenting cells (APCs) that playing a critical role between innate and adaptive immune response [38]. However, we found some adapt immune related cells (activated T, B cells) did not appear to increase, indicating that DCs may activate inflammation through innate immune pathways. The research on DCs in FM is very rare, but some progress has been made in other diseases. In diabetic atherosclerosis, Zhao et al. reported that DCs could mediate the chronic low-grade inflammation by the RAGE-TLR4-PKCβ1 signaling pathway [39]. In inflammatory bowel disease, activated DCs could release the inflammatory cytokines such as IL-6, IL-12 and tumor necrosis factor (TNF-α) by up-regulating the pattern recognition receptors [Toll-like receptor (TLR) 2 and TLR4], MHC class II molecules, and costimulatory molecules (CD40 and CD86) [40]. Therefore, we speculated that the inflammation in FM may be related to the innate immune response activated by DCs. The functional enrichment analyses also showed the innate immune response were significantly enriched. The antibacterial response was upregulated in micro-inflammation group, suggesting that the inflammatory pathway in FM might overlap to some extent with the antibacterial response pathway in the human body. Most DEGs of this term, including *DEFA4*, *DEFA3*, *LTF*, *BPI*, and *RNASE3*, were closely associated with neutrophils, indicating that the neutrophils were activated to some extent in micro-inflammation, although their proportion in peripheral blood did not increase. Neutrophil dysregulation enriched by Reactome pathways analyses also confirmed our speculation. However, further research is still needed to investigate the deeper mechanisms and the formation of inflammation, especially neuroinflammation, in FM.

### How do we distinguish inflammatory FM?

The different subtypes of FM have different clinical characteristics, immune features and pathways activities. Therefore, it is necessary to construct an accurate classifier to distinguish between the two subtypes in order to help clinical doctors implement precision treatment. In recent years, machine learning has been increasingly used in disease diagnosis due to its high accuracy [41, 42]. The SVM method based on statistical learning theory is considered the best method for small-sample classification and regression problems because it solves practical problems such as nonlinearity, high dimensions, and local minima quite well [22, 43]. In this study, we firstly performed the feature selection to screen the most representative DEIGs. Lasso regression, RF-RFE and XGBoost were all widely used methods with their own advantages. After joint analysis, 5 DEIGs were selected. The two significant clinic characteristics, depression and BMI, were also included. SVM was then used to construct a classifier. The internal validation revealed that this classifier had high precision with AUC of 0.945, sensitivity of 0.941 and specificity of 0.929. The Hosmer–Lemeshow goodness-of-fit test exhibited satisfactory concordance between predicted and actual outcomes (*p* = 0.832). These findings implied that 5 DEIGs might be key biomarkers to identify micro-inflammation and non-inflammation patients. Unfortunately, due to the lack of more sequencing data of FM, we cannot obtain more samples for external validation.

However, there are several limitations in our research. For example, our research was a bioinformatic analysis based on microarray data of small samples, which may result in false negatives or false positives. Due to the partial lack of clinical information in GSE67311, we are unable to further analyze the differences between ages, disease courses, and complications such as insomnia between different subtypes. Our next step will be to expand the sample size for further verification and research.

In conclusion, we identified the micro-inflammation subtype of FM patients, which have more depression, higher BMI, and more activated dendritic cells and neutrophils. We also developed a new classification system to help clinical doctors identify the different subtypes in FM and implement precision treatment. The specific potential mechanism between inflammatory response-related genes and FM, however, remains unclear, and further investigation into the subject may prove useful to understanding FM.

### Supplementary Information

**Additional file 1: Table S1.** The gene sets for marking 28 immune cell types. **Table S2.** The differentially expressed genes between fibromyalgia and control group. **Table S3.** The inflammatory genes in GO 0006954 term. **Table S4.** The differentially expressed genes between C1 and C2 group.

## Data Availability

All data used to support the findings of this study are included within the article. The datasets used and analyzed during the current study are available from GSE67311 (http://www.ncbi.nlm.nih.gov/geo). Further inquiries can be directed to the corresponding author.

## Abbreviations

FM: Fibromyalgia
GEO: Gene Expression Omnibus database
DEIGs: Differentially expressed inflammatory genes
DEGs: Differentially expressed genes
SVM: Support vector machine
ssGSEA: Single-sample gene-set enrichment analysis
GO: Gene Ontology
MF: Molecular function
BP: Biological process
CC: Cellular component
KEGG: Kyoto Encyclopedia of Genes and Genomes
DAVID: The Database for Annotation, Visualization and Integrated Discovery
LASSO: Least Absolute Shrinkage and Selection Operator
RF-RFE: Random Forest Recursive Feature Elimination
XGBoost: EXtreme Gradient Boosting
ROC: Receiver operating characteristic
AUC: Area under curve
NK cells: Nature killer cells
DCs: Dendritic cells
APCs: Antigen presenting cells
TNF-α: Tumor necrosis factor
FIQR: Fibromyalgia Impact Questionnaire Revised
